# An atypical case of iatrogenic LAD dissection following PCI: Diagnostic and management challenges

**DOI:** 10.1016/j.radcr.2023.09.085

**Published:** 2023-10-20

**Authors:** Renato Fabrizio, Laura Mascitti, Carmela Furci

**Affiliations:** Postgraduation School in Radiodiagnostics, Università degli Studi di Milano, Via Festa del Perdono 7, 20122, Milano MI, Italy

**Keywords:** Cardiac CT, Cardiovascular imaging, Coronary artery, Iatrogenic coronary artery dissection, PCI

## Abstract

Iatrogenic coronary artery dissection is listed among the rare complications of interventional cardiology procedures, such as diagnostic angiography and percutaneous coronary intervention (PCI), which are worldwide routine in clinical practice.

Although extremely infrequent, they can lead to severe myocardial injury and even to death.

In the following case, we present an iatrogenic left anterior descending coronary artery (LAD) dissection, which led to an ischemic dilated cardiopathy.

This case was radiologically and clinically worthy of note for the atypical site of dissection, the lack of symptoms although the ischemic damage and the global management requiring a multidisciplinary approach.

## Introduction

Iatrogenic coronary artery dissection is a recognized rare but potentially fatal complication of percutaneous coronary intervention (PCI), caused by disruption of the coronary intima by mechanical trauma due to strong manipulation of rigid guide wires and vigorous contrast agent injection [1].

Considering the worldwide high prevalence of PCI, incidence of dissection is very low occurring in 0.02%-0.07% cases and it mainly involves the right coronary artery and the left main coronary artery [2,3].

Although the global incidence is low, dissection can lead to procedure failure, increased risk of type 4a myocardial infarction and periprocedural myocardial injury due to abrupt vessel closure [4].

The management can be either conservative, surgical or interventional with a revascularization procedure depending on clinical, biochemical, and imaging information.

We reported this case because it shows an atypical presentation of coronary artery dissection leading to an ischemic cardiopathy and it demonstrates how imaging is fundamental diagnosis and complications prevention.

## Case report

A 55-year-old woman performed a cardiological examination for asthenia and intolerance to stress. The patient had no cardiovascular risk factors except for a left branch block known from the age of 29.

The cardiologist prescribed a transthoracic echocardiography, showing an ejection fraction (EF) of the left ventricle (LV) of 35%-40% with mild LV inferior wall hypokinesia, and an electrocardiogram Holter, showing frequent premature ventricular beats (PVBs) and supraventricular tachycardia (SVT). Blood chemistry tests were normal.

In order to establish the cause of these new-found disorders, the patient was subjected to an elective coronary angiography, which was unremarkable.

**Fig. 1:**
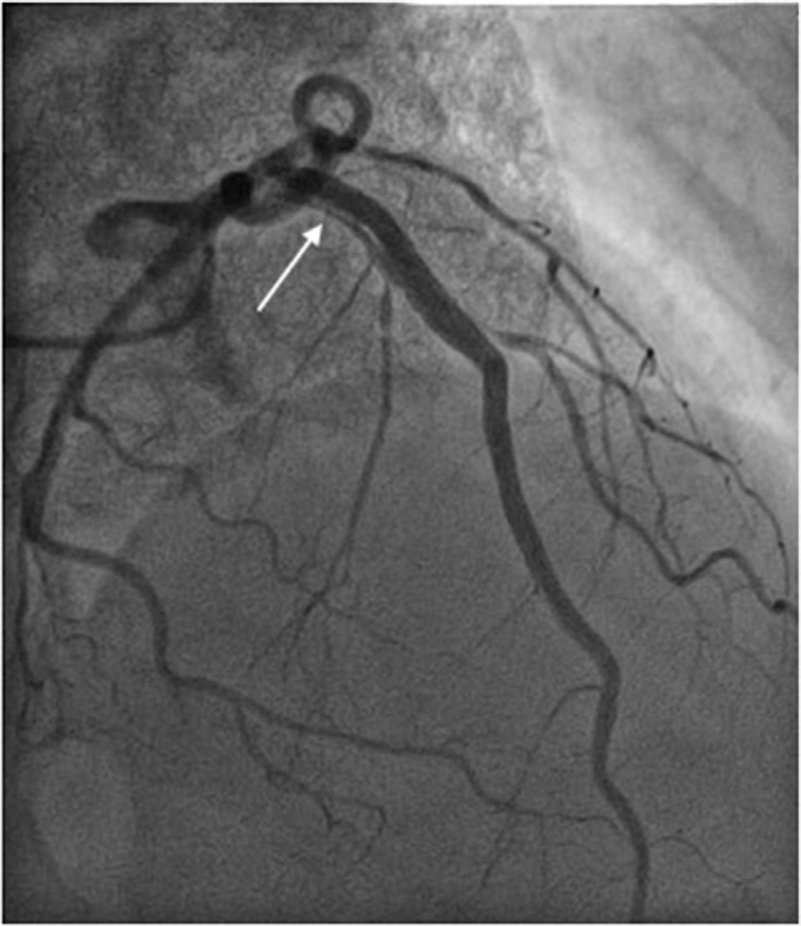
Coronary angiography showing dissection of the left anterior descending artery. The white arrow shows the late opacification of the false lumen by the contrast agent compared to the strong opacification of the true lumen. The linear radiolucency represents the intimal flap.

The night after the procedure, the patient complained of chest pain and significant increase in troponins; therefore, another urgent coronary angiography was performed, showing a dissection of the left anterior descending artery (LAD) in the paraostial, proximal and middle third (Fig. 1).

The patient was transferred for intensive monitoring to the coronary unit and a protective cardiotherapy with betablocker, ACE inhibitor, cardioaspirin, and mineralcorticoid receptor antagonist has been set.

Clinical conditions improved in a few days and the medical team decided for a conservative approach.

The patient was discharged with prescriptions for transthoracic echocardiography, coronary CT, and cardiological control examination within 1 month.

**Fig. 2:**
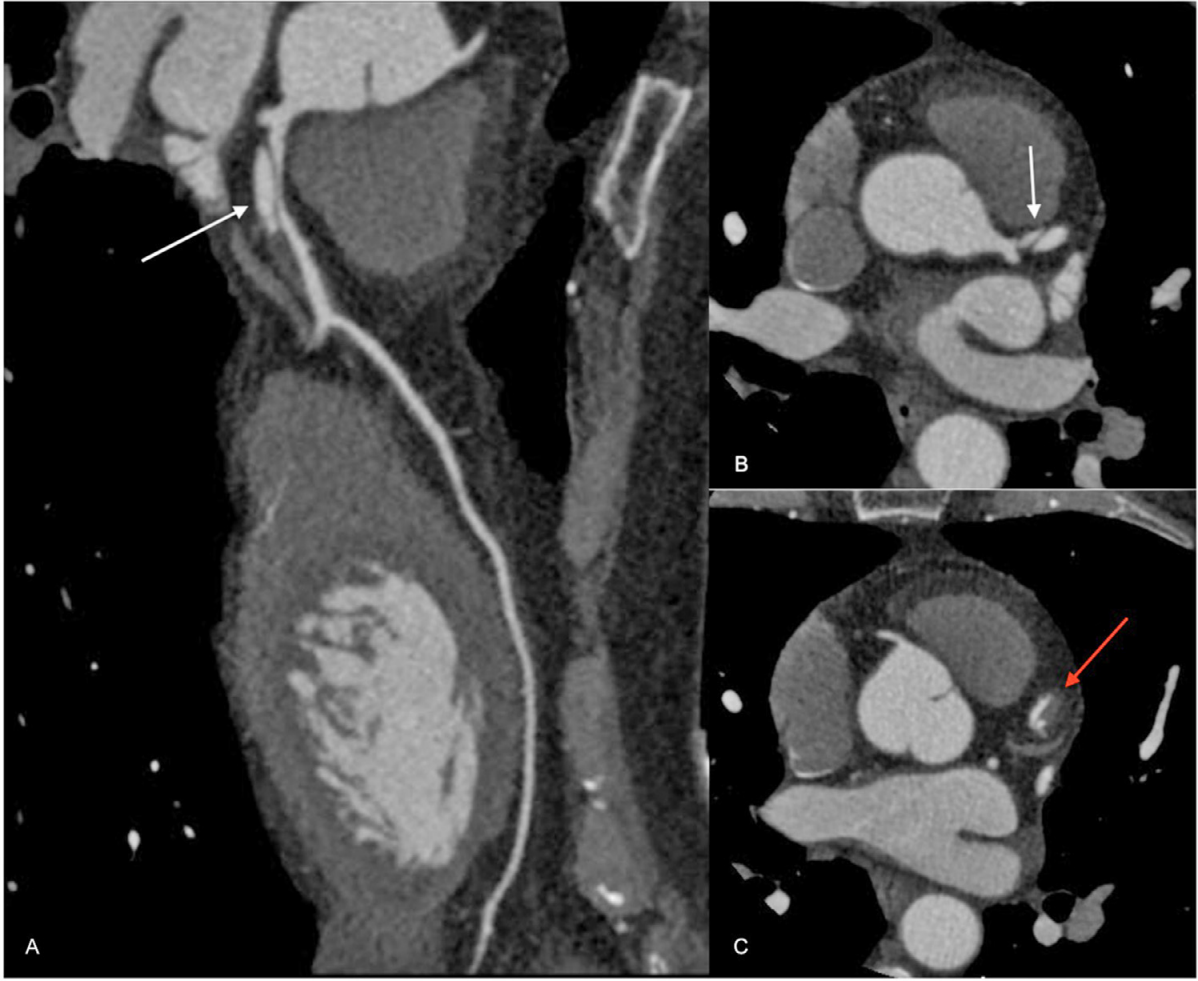
Coronary CT scan images showing dissection of LAD. The white arrows in the oblique (A) and in the axial (B) images focus on the dissection flap of the proximal and middle tract of the vessel causing narrow of the true lumen, whereas the red arrow in the second axial image (C) shows the partial thrombosis of the false lumen.

**Fig. 3:**
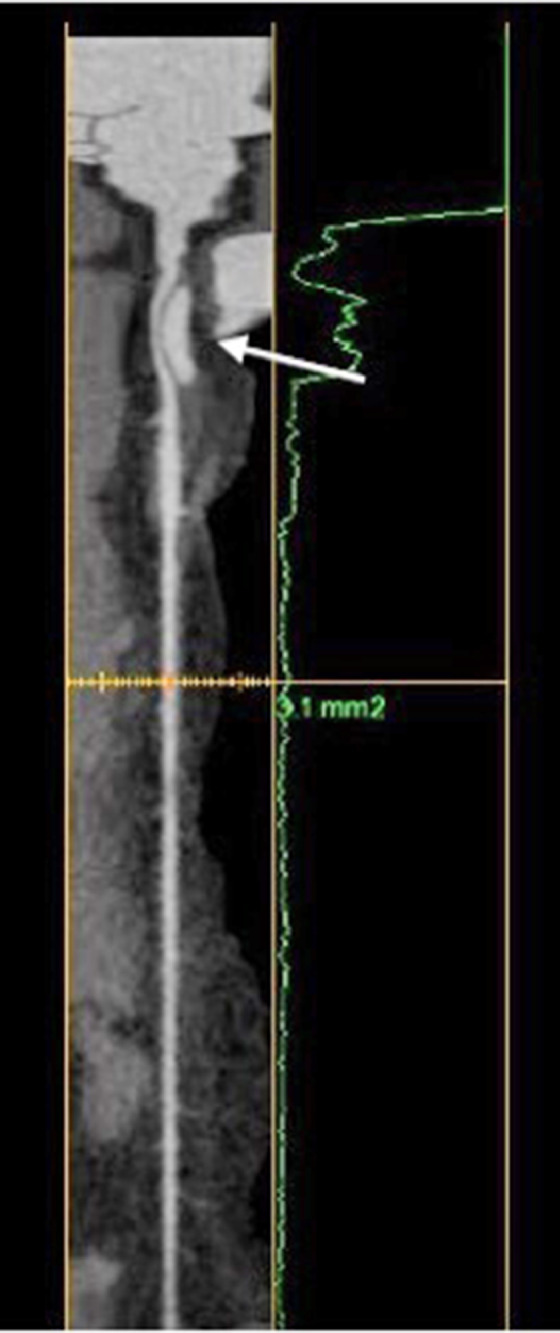
Lumen reconstruction of enhanced CT scan showing the left anterior descending artery in its entire length. The white arrow indicates the dissected tract.

The subsequent coronary CT revealed a narrowing of the true lumen in the proximal tract of the LAD due to compression by the false lumen (Figs. 2 and 3).

Moreover, the new transthoracic echocardiography demonstrated akinesia of the cardiac apex, of the para-apical segments, of the septum and of the basal infero-posterior wall of the left ventricle, with a moderate systolic dysfunction (EF 38%).

For these reasons, the treating cardiologist decided to submit the patient to a cardiac MRI, which showed a mild dilatation on the LV with hypo-akinesia of the anterior wall in the middle area-apex of the LV and, most important, multifocal areas of subendocardial late enhancement after contrast agent injection involving basal to mid anterior/anteroseptal segments of the LV, reported as an ischemic pattern.

Considering these findings, the treating cardiologist and the interventional cardiologist decided to treat the patient with a new PCI with a drug-eluting stent (DES) in the proximal and middle tract of LAD.

The patient was discharged in a few days in good clinical conditions, with the cardiological therapy previously reported and with clinical and imaging follow-up 6 months apart.

## Discussion

Iatrogenic coronary artery dissection is an uncommon but important complication of PCI; most frequently, acute stent thrombosis, stroke, vascular access bleeding or increases in cardiac biomarkers are reported.

Among all coronary artery dissections, iatrogenic LAD dissection is an extremely rare event.

As Ramasamy et al.[5] reported in their study, the right coronary artery is involved up to 50% of cases, followed by the left main stem (LMS) in 46% of cases; both arteries are most frequently dissected during PCI that during diagnostic coronary angiography (25%-30% against 75%-80% respectively).

Considering PCI as the principal revascularization strategy for patients with obstructive coronary artery disease with 5 million procedures performed worldwide each year [4], death after diagnostic cardiac catheterization occurs only in 0,071% of cases and dissection is the principal cause in up to 67% of cases [6].

Coronary artery dissections can be classified as types A to F based on the National Heart, Lung and Blood Institute (NHLBI) system developed from the Coronary Angioplasty Registry depending on the angiographic appearances of the intimal disruption and contrast clearance [5].

As Cheng et al. [3] and Rogers et al. [7] said, PCI represent the best treatment option for larger dissections with or without hemodynamic instability, in order to avoid acute vessel closure, which can lead to myocardial infarction and even to death.

In the case reported, the dissection was initially mild although extensive, with no involvement of the LMS and with good patient's clinical conditions; therefore, it has been decided for a conservative approach.

But as shown in the further examinations, the dissection turned to be flow-limiting with a significant narrowing of the LAD lumen (Figs. 2 and 3).

**Fig. 4:**
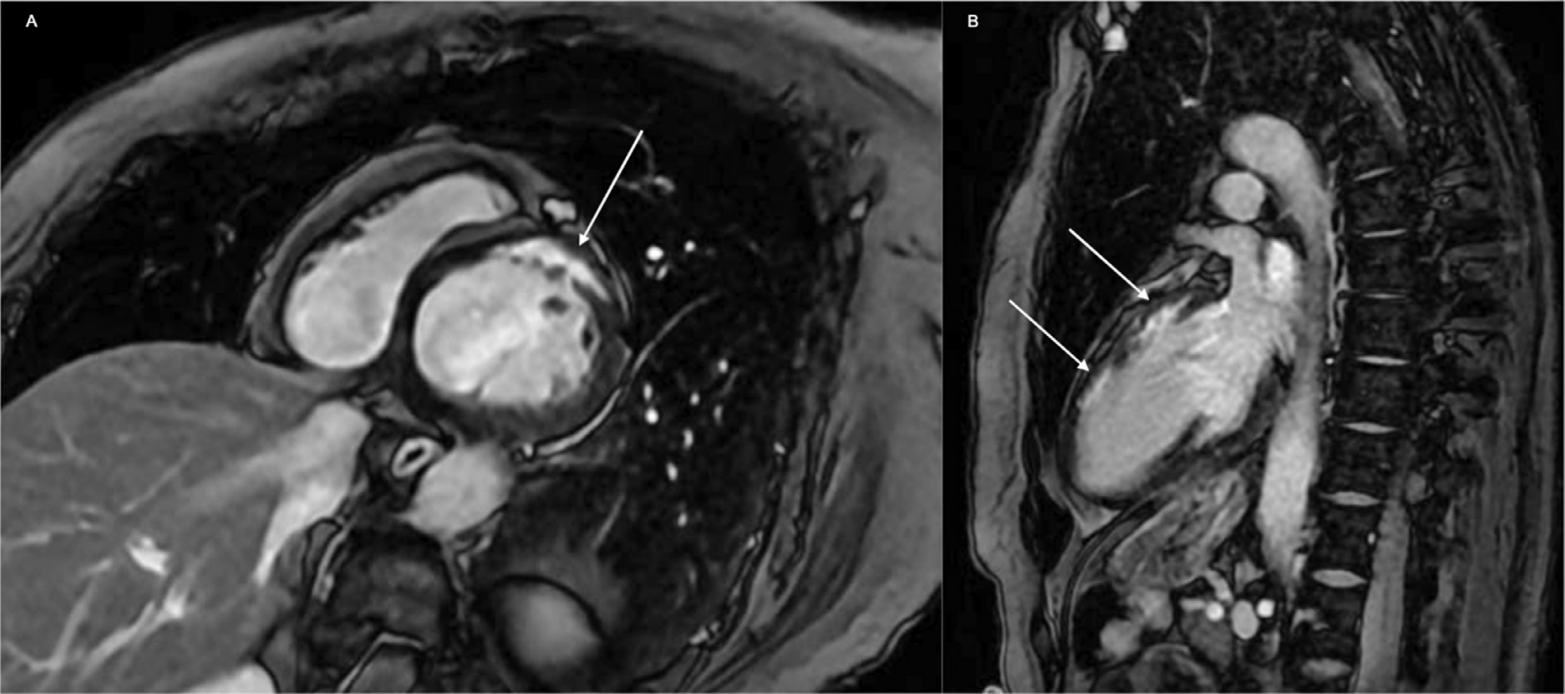
Cardiac MRI short axis (A) and 2-chamber (B) images of a bSSFP (balanced steady-state free precession) sequence acquired 10 minutes after contrast agent injection. The white arrows show the area of subendocardial late enhancement, involving the anterior wall of the left ventricle with a typical ischemic pattern.

That led to an ischemic damage of the myocardium (Fig. 4) with kinetic and functional alterations of the LV, fulfilling the definition of procedural myocardial infarction (type 4a) by the fourth universal definition of myocardial infarction (2018), which is defined by cardiac biomarker elevations with evidence of new myocardial ischemia [4].

Imaging had a key role: the coronary CT highlighted the anatomical complications, while the MRI delineated the extent of myocardial injury.

In this case, imaging was fundamental in decision-making, because it enabled clinicians to recognize the specific issue of the patient, reducing time-wasting and guaranteeing better outcome.

## Conclusion

This case underscores how rare and atypical LAD dissection is and how prompt recognition and accurate management are key points for favorable early and long-term outcome [8].

Advanced imaging modalities have an invaluable role not just in diagnosis but also in guiding therapeutic management.

In fact, combining imaging information with clinical and biochemical information enable to an overall evaluation of patients, preventing from severe myocardial damage.

## Patient consent

Written, informed consent was obtained from the patient for publication of this case.
